# Identification and Validation of Circulating Micrornas as Prognostic Biomarkers in Pancreatic Ductal Adenocarcinoma Patients Undergoing Surgical Resection

**DOI:** 10.3390/jcm9082440

**Published:** 2020-07-30

**Authors:** Natalia Gablo, Karolina Trachtova, Vladimir Prochazka, Jan Hlavsa, Tomas Grolich, Igor Kiss, Josef Srovnal, Alona Rehulkova, Martin Lovecek, Pavel Skalicky, Ioana Berindan-Neagoe, Zdenek Kala, Ondrej Slaby

**Affiliations:** 1 Central European Institute of Technology, Masaryk University, 625 00 Brno, Czech Republic; gablon@wp.pl (N.G.); trachtova@mail.muni.cz (K.T.); 2 Department of Surgery, Faculty Hospital Brno and Faculty of Medicine, Masaryk University, 601 77 Brno, Czech Republic; Prochazka.Vladimir@fnbrno.cz (V.P.); Hlavsa.Jan@fnbrno.cz (J.H.); Grolich.Tomas@fnbrno.cz (T.G.); 3 Masaryk Memorial Cancer Institute, Department of Comprehensive Cancer Care, 602 00 Brno, Czech Republic; kiss@mou.cz; 4 Laboratory of Experimental Medicine, Institute of Molecular and Translational Medicine, Faculty of Medicine and Dentistry, Palacky University and University Hospital Olomouc, 771 47 Olomouc, Czech Republic; josef.srovnal@fnol.cz (J.S.); alona.rehulkova@upol.cz (A.R.); 5 1st Department of Surgery, Faculty of Medicine and Dentistry, Palacky University and University Hospital 771 47 Olomouc, Czech Republic; Martin.Lovecek@fnol.cz (M.L.); pavel.skalicky@upol.cz (P.S.); 6 MEDFUTURE-Research Center for Advanced Medicine, University of Medicine, and Pharmacy Iuliu-Hatieganu, 400000 Cluj-Napoca, Romania; ioana.neagoe@umfcluj.ro; 7 Department of Pathology, Faculty Hospital Brno and Faculty of Medicine, Masaryk University, 625 00 Brno, Czech Republic; 8 Department of Biology, Faculty of Medicine, Masaryk University, 625 00 Brno, Czech Republic

**Keywords:** Pancreatic ductal adenocarcinoma, prognosis, microRNAs

## Abstract

Pancreatic ductal adenocarcinoma (PDAC) is one of the most lethal and aggressive cancers with a less than 6% five-year survival rate. Circulating microRNAs (miRNAs) are emerging as a useful tool for non-invasive diagnosis and prognosis estimation in the various cancer types, including PDAC. Our study aimed to evaluate whether miRNAs in the pre-operative blood plasma specimen have the potential to predict the prognosis of PDAC patients. In total, 112 PDAC patients planned for surgical resection were enrolled in our prospective study. To identify prognostic miRNAs, we used small RNA sequencing in 24 plasma samples of PDAC patients with poor prognosis (overall survival (OS) < 16 months) and 24 plasma samples of PDAC patients with a good prognosis (OS > 20 months). qPCR validation of selected miRNA candidates was performed in the independent cohort of PDAC patients (*n* = 64). In the discovery phase of the study, we identified 44 miRNAs with significantly different levels in the plasma samples of the group of good and poor prognosis patients. Among these miRNAs, 23 showed lower levels, and 21 showed higher levels in plasma specimens from PDAC patients with poor prognosis. Eleven miRNAs were selected for the validation, but only miR-99a-5p and miR-365a-3p were confirmed to have significantly lower levels and miR-200c-3p higher levels in plasma samples of poor prognosis cases. Using the combination of these 3-miRNA levels, we were able to identify the patients with poor prognosis with sensitivity 85% and specificity 80% (Area Under the Curve = 0.890). Overall, 3-miRNA prognostic score associated with OS was identified in the pre-operative blood plasma samples of PDAC patients undergoing surgical resection. Following further independent validations, the detection of these miRNA may enable identification of PDAC patients who have no survival benefit from the surgical treatment, which is associated with the high morbidity rates.

## 1. Introduction

Pancreatic ductal adenocarcinoma (PDAC) constitutes 90% of all pancreatic cancers and is associated with the worst survival, with only 5–7% of patients living longer than five years from diagnosis [1,2]. In comparison to other types of pancreatic cancer [3], PDAC, due to its aggressive biological behavior, has a high incidence/mortality ratio reaching 94%, which makes this disease the 4th most common cause of cancer-associated mortality in developed countries [2]. Among the available treatment strategies, including radical pancreatectomy, chemotherapy, radiotherapy, or integrated multimodal treatment, radical surgical resection of the tumor in its early stages (IA–IIB) remains the only option that may increase the five-year survival rate to 16–21% [4,5,6]. Nevertheless, most of the patients are diagnosed at an advanced inoperable stage of the disease, and only 15–20% of PDAC patients can be considered candidates for radical surgical resection. Clinical studies revealed that there is a subset of PDAC patients, who develop disease recurrence shortly after resection without any improvement in survival [7,8,9]. Compared to PDAC patients with inoperable disease receiving only chemotherapy, this subset of poor prognosis patients has no clinical benefit from the surgical resection, which is associated with a high morbidity rate. Therefore, pre-operative estimation of the prognosis is essential to avoid treatment-related risks for those patients who would unlikely benefit from this treatment approach [10,11]. One of the reasons behind poor outcomes is the presence of micro-metastatic disease, which cannot be detected during pre-operative staging examination and even during intraoperative assessment [10]. Unfortunately, there is still a lack of powerful biomarkers, that would contribute to better individualization of PDAC patients’ treatment strategies. Currently, all PDAC patients with localized disease and operable tumors evaluated through imaging methods are candidates for surgical treatment [12]. Carbohydrate antigen 19-9 (CA19-9) is the only approved blood-based biomarker used in PDAC patients, but it is mainly useful for monitoring of PDAC patients following the surgical resection rather than for pre-operative survival prognostication [13]. In the last few years, there has been increasing evidence suggesting circulating cell-free microRNAs (miRNAs) present promising non-invasive biomarkers in cancer. 

MiRNAs are highly conserved, small, non-coding RNAs, 18–25 nucleotides in length. miRNAs are involved in post-transcriptional regulation of gene expression. miRNAs act as powerful regulators of gene expression, mostly via interacting with the 3′ UTR of target mRNAs, which inhibit translation or induces mRNA degradation [14]. Additionally, they can also bind to 5’UTR of target mRNAs or bind to coding sequence regions [15]. A number of studies have revealed that miRNAs are involved in the regulation of cell homeostasis by controlling important cellular processes including the development, differentiation, proliferation, apoptosis and stress reaction. Furthermore, aberrant expression of miRNAs can promote carcinogenesis through direct or indirect regulation of oncogenes or tumor suppressor genes [16]. 

In PDAC, many investigators have detected changes in miRNA expression patterns, which influenced multiple genetic aberrations that contribute to initiation of tumorigenesis progression, invasion, and metastatic processes. Subsequently, miRNAs correlate with disease-free, overall survival of PDAC patients, and their response to treatment [17]. 

Interest in the minimally invasive biomarkers is growing because they would significantly contribute to improving the outcome of PDAC patients by individualization of the therapeutic approach. The presence of tumor-derived miRNAs in body fluids offers an opportunity to obtain such biomarker and became the subject of intense investigation. miRNAs are selectively and specifically released into circulation under various pathological conditions including cancer. Moreover, circulating miRNAs present many favorable advantages for application as liquid biopsy-based biomarkers, such as high stability, high abundance, and their presence in nearly all body fluids including blood plasma [18,19,20].

The aim of our study was to enable identification of the PDAC patients who will not benefit from the surgical resection (patients with the same or shorter overall survival (OS) as PDAC patients without surgical resection) and therefore give the rationale for the clinical decision, whether to perform surgical resection or give preference to non-surgical therapeutic modalities and quality of life.

To this end, we performed global profiling of blood plasma miRNAs using small RNA sequencing followed by the validation of miRNA candidates in the independent cohort of PDAC patients to assess the potential of plasma miRNAs for pre-operative prognostic stratification of PDAC patients planned for radical surgical resection. 

## 2. Material and Methods

### 2.1. Study Population Characteristic

Treatment-naive patients with histologically confirmed pancreatic ductal adenocarcinoma were recruited from the University Hospital Brno (UHB; Brno, Czech Republic), University Hospital Olomouc (UHO; Olomouc, Czech Republic), and Masaryk Memorial Cancer Institute (MMCI; Brno, Czech Republic). Only patients undergoing radical surgical resection were enrolled in our study. Subjects were of the same ethnicity (Caucasian). Patient characteristics are summarized in Table 1. The study was approved by multi-centric Ethical Board (UHB) and written informed consent was provided by each study participant. Approximately 10 mL of peripheral venous blood was collected in an ethylenediaminetetraacetic acid (EDTA)-treated Vacutainer before surgical resection and any other treatment. Within one hour after collection, plasma fraction was separated by centrifugation at 1200× *g* for 10 min at 4 °C and stored at −80 °C till further processed. 

#### 2.1.1. RNA Isolation from Blood Plasma Specimens

Cell-free miRNAs were isolated from 250 µL of blood plasma using Qiagen miRNeasy Serum/Plasma Kit (Qiagen, GmbH, Hilden, Germany) according to manufacturers’ protocol. We used glycogen during isolation step as an RNA carrier, since exogenous RNA can interfere—via non-specific hybridization or amplification—with the results of small RNA sequencing [21]. Concentration and purity of RNA was measuring by both UV spectrophotometry (NanoDrop ND-2000, Thermo Fisher Scientific, Walthman, MA, USA) and fluorometry (Qubit, Thermofisher Scientific, Walthamn, MA, USA). The purified RNA was stored at −80 °C until further analysis or processed immediately. 

#### 2.1.2. Small RNA Libraries Preparation and Next Generation Sequencing

In the discovery phase of the study, we used a blood plasma samples collected pre-operatively from PDAC patients, which have been already available in the biobank of Masaryk Memorial Cancer Institute (Brno, Czech Republic). For small RNA sequencing, libraries were prepared from 2 µL of total RNA using the QIAseq™ miRNA Library Kit and QIAseq miRNA NGS 48 Index IL (Qiagen, Hilden, Germany). Following 3′ and 5′ adapter ligation, small RNAs were reverse transcribed, using unique molecular identifier (UMI) primers of random 12-nucleotide sequences. This way, precise linear quantification of miRNAs is achieved, overcoming potential PCR-induced biases. cDNA libraries were amplified by PCR for 24 cycles, with a 3′ primer that includes a 6-nucleotide unique index. Following size selection and cleaning of the sequencing libraries with magnetic beads, quality control was performed by measuring library concentration with a Qubit fluorometer using a dsDNA High Sensitivity (HS) assay kit (Thermo Fisher Scientific, Walthamn, MA, USA) and confirming library size with TapeStation D1000 (Agilent). Further, libraries were multiplexed and sequenced on a single NextSeq 500/550 v2 flow cell (Illumina, San Diego, CA, USA) with 75bp single read and 6bp index read (80 cycles). 

#### 2.1.3. Quantitative Real-Time PCR (qRT-PCR)

In the validation phase of the study, we used a prospectively collected plasma samples withdrawn pre-operatively from PDAC patients at University Hospital Brno (Brno, Czech Republic) and University Hospital Olomouc (Olomouc, Czech Republic). For miRNA qRT-PCR, plasma RNA was reverse-transcribed to complementary DNA (cDNA) using a miRCURY LNA Universal RT cDNA Synthesis Kit (Qiagen, Hilden, Germany). Initial RNA input used in reverse transcription reaction was optimized by running a few individual assays with different volumes of RNA samples according to manufacture requirements. We used 2 µL of RNA template in 10 µL of final reaction volume for all samples, which correspond to 16 µL of original blood plasma specimens. Reverse Transcription was performed using T100™ Thermal Cycler (Bio-Rad, Hercules, CA, USA) at 42 °C for 60 min and 90 °C for 5 min. The final product of reaction was diluted in ratio 1:30 with nuclease free water. Three microliters of diluted cDNA were added to qPCR mixture (miRCURY SYBR Green PCR Kit) containing LNA-enhanced primers specific for each miRNA (miRCURY LNA miRNA PCR primers; hsa-miR-9-5p; hsa-miR-30e-5p; hsa-miR-365a-3p; hsa-miR-22-3p; hsa-miR-885-5p; hsa-miR-99b-5p; hsa-miR-99a-5p; hsa-miR-200c-3p; hsa-miR-122-5p; hsa-miR-100-5p; hsa-let-7e-5p; hsa-miR-93-5p). All samples were run using Quant Studio 12K Flex Sstem (Applied Biosystems, Foster City, CA, USA).

#### 2.1.4. Data Normalization and Statistical Analysis

The raw sequencing images from Illumina NextSeq 550 (change for the right machine used) were demultiplexed and converted to fastq format using bcl2fastq (version 2.20.0). Adapter sequences in raw sequencing data were identified by Kraken package (15-065) and trimmed using Cutadapt (version 1.18). Collapsing was performed utilizing unique molecular identifiers (UMIs) with FASTX-Toolkit (version 0.0.14). Subsequently, reads were quality trimmed and these shorter than 15bp were discarded. Reads originating from snoRNAs, snRNAs, rRNAs, tRNAs, piRNAs, and YRNAs (downloaded from Ensembl and RefSeq) were identified using Bowtie (version 1.2.2) and removed from the data. Remaining reads were mapped against the miRBase (version 21) using the miraligner tool (version 1.2.4). Statistical analysis, including normalization for library depth, was carried out in R (version 3.4.3) with DESeq2 package (version 1.18.1).

The threshold cycle data were calculated by QuantStudio 12K Flex software. All real-time PCR reactions were run in duplicates. All PCR reactions where the difference between Ct values in duplicate were higher than 0.25 were repeated. The average expression levels of all measured miRNAs were normalized using miR-93-5p which was found to be suitable reference gene based on consensus of two algorithm, namely NormFinder and geNorm. Quantification of target miRNA relative to reference endogenous control was determined by the 2^−ΔCt^ method [22]. Statistical differences between the levels of analyzed miRNAs in plasma samples of poor and good prognosis cases were evaluated by two-tailed non-parametric Mann–Whitney U-test. Further, receiver operating curve (ROC) analyses were performed to assess the diagnostic performance of analyzed miRNAs. Survival analyses were carried out using the log-rank test and Kaplan–Meier analysis. All calculations were performed using GraphPad Prism version 7.01 (GraphPad Software) *p*-values of less than 0.05 were considered statistically significant.

## 3. Results

In total, 112 patients with PDAC were enrolled in the study. These patients were divided into two cohorts: discovery (*n* = 48) and validation (*n* = 64) cohort. In terms of prognosis, patients with overall survival (OS) shorter than 16 months were classified as poor prognosis cases, conversely patients with OS longer than 20 months (without event) were classified as good prognosis cases. According to this definition of prognostic groups, there was 24 good prognosis and 24 poor prognosis cases in the discovery cohort and 16 good prognosis and 14 poor prognosis cases in the validation cohort. Remaining 34 cases in the validation cohort were included in the survival analysis but were not used for the group analysis due to the short follow-up or intermediate OS between the good and poor prognosis survival ranges.

In this discovery phase of the study, we performed small RNA sequencing of the pre-operative blood plasma specimens, and we identified 44 miRNAs to have significantly different levels in the plasma samples of the 24 patients with good prognosis cases and 24 with poor prognosis (*p* < 0.05). Among these miRNAs, 21 showed higher expression and 23 showed lower expression in blood plasma from PDAC patients with poor prognosis (Table 2). Out of these 44 miRNAs identified in discovery phase, 11 miRNAs (miR-99a-5p, miR-9-5p, miR-365a-3p, miR-22-3p, miR-885-5p, miR-200c-3p, let-7e-5p, miR-100-5p, miR-122-5p, miR-99b-5p, miR-30e-5p) were selected for the validation phase of the study to evaluate their ability to distinguish PDAC patients with poor and good prognosis. These miRNAs were selected based on the *p*-value (*p* < 0.02), log2(fold-change) ≥ 0.45 or ≤−0.45, and the average number of reads across all sequenced samples (at least 10). Lower threshold for the fold-change was selected based on the pleiotropic regulatory effects of miRNAs and related biological relevance of even subtle expression changes compared to the mRNAs.

**Figure 1:**
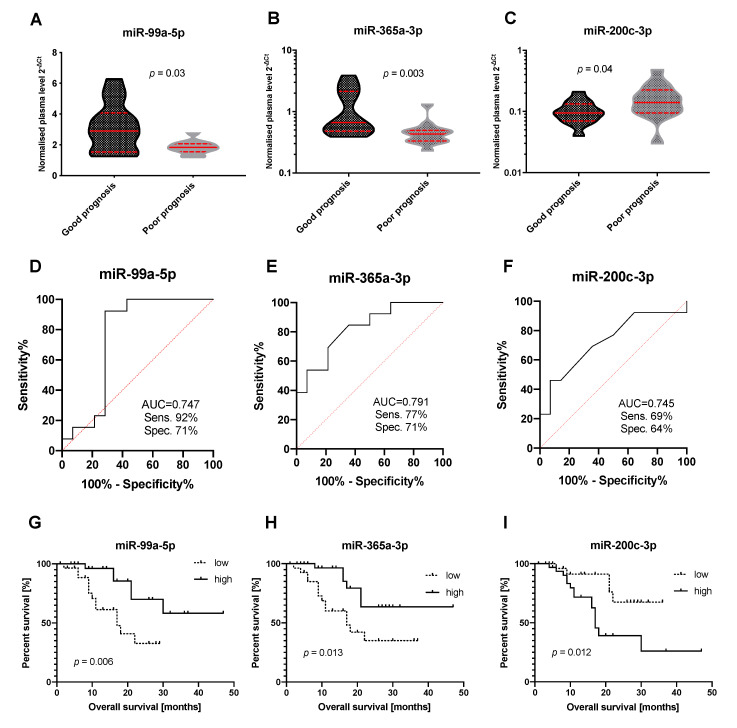
miRNAs with significantly different levels in blood plasma of pancreatic ductal adenocarcinoma (PDAC) patients with good and poor prognosis after surgical resection. Results of Mann–Whitney U-test for miR-99a-5p (**A**), miR-365a-3p (**B**), and miR-200c-3p (**C**); ROC analysis for miR-99a-5p (**D**), miR-365a-3p (**E**) and miR-200c-3p (**F**); and Kaplan-Meier survival analysis for miR-99a-5p (**G**), miR-365a-3p (**H**) and miR-200c-3p (**I**). AUC: Area under the curve; Sens.: Sensitivity; Spec.: Specificity.

The blood plasma levels of miRNA candidates from the discovery cohort were determined by use of individual qPCR assays and statistically evaluated between the groups of patients with good and poor prognosis. Using two-tailed non-parametric Mann–Whitney U test and ROC analysis, only miR-99a-5p and miR-365a-3p were confirmed to have significantly lower levels and miR-200c-3p significantly higher levels in the blood plasma of the patients with poor prognosis (*p*-values and AUC values are summarized in Table 3 and Figure 1A–F). Other tested miRNAs were not confirmed to have different levels in blood plasma samples of PDAC patients with different prognosis. These results were also confirmed using the Kaplan–Meier analysis. Patients with lower levels of miR-99a-5p and miR-365a-3p and higher levels of miR-200c-3p in blood plasma had significantly shorter overall survival (Figure 1D–I).

**Figure 2:**
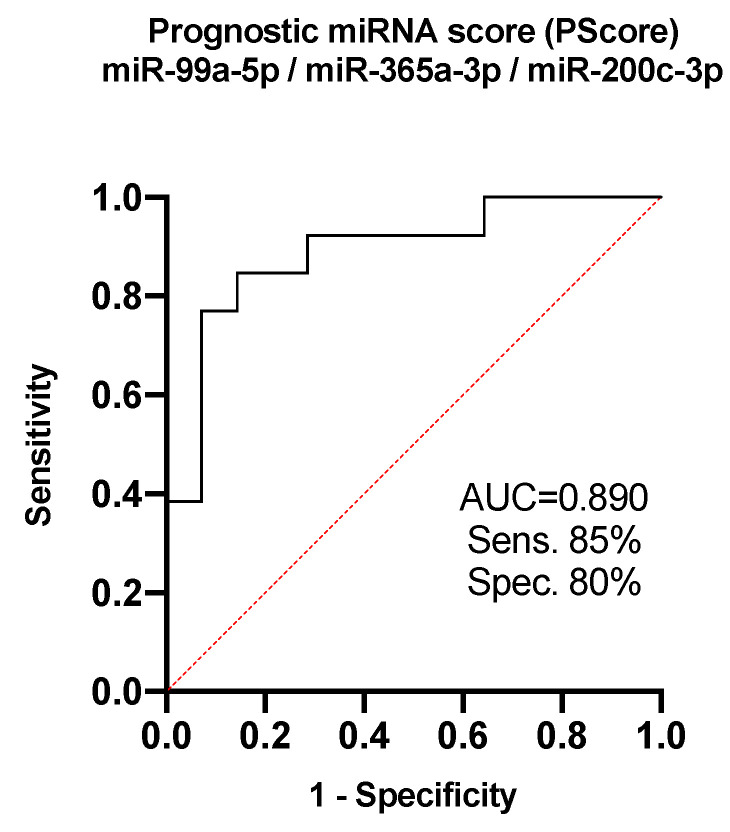
Receiver operating characteristic analysis of the use of miR-99a-5p, miR-365a-3p, and miR-200c-3p combination (Pscore) in the discriminating between the PDAC patients with good and poor prognosis. AUC: area under the curve.

Subsequent ROC analysis revealed that the usage of 3-miRNA-combined prognostic score (PScore = −0.6430 + 0.8689 *miR-99a-5p + 0.9261 *miR-365a-3p-17.5256 *miR-200c-3p), as established by a bidirectional stepwise logistic regression, enabled the identification of the patients with poor prognosis after surgical resection (OS < 16 months) with sensitivity of 85% and specificity of 80% (area under the curve (AUC) = 0.890; cut-off value = 0.5522; Figure 2). Prognostic score based on the combination of three miRNAs enabled us to increase the AUC from 0.791, which was the highest in reached by individual miRNA, to 0.890.

## 4. Discussion

Complete removal of the tumor at its early stage is considered only as an curative option for PDAC patients. However, a significant percentage of PDAC patients that undergo primary tumor resection rapidly develop a disease recurrence, whereas other patients benefit from surgery and have long disease-free survival. Identifying the patients at risk of early disease recurrence could enable adjustments in rational treatment selection to perform surgical resection or give preference to non-surgical treatment modalities and quality of life. There is increasing evidence that cell-free miRNAs are suitable candidates for the prediction of PDAC progression due to their altered expression during tumorigenesis and their stability in the body fluids [19,23].

Herein, we present results of a multicenter study where we sought to identify circulating cell-free miRNAs with the potential of pre-operative prognostic stratification of PDAC patients in a minimally invasive way. For this purpose, we implemented a small RNA sequencing technique for global miRNA in pre-operative plasma samples from treatment-naive patients with PDAC at the operable stage of the disease. Patient populations in both the discovery and validation cohorts were divided into two prognostic groups according to their survival after curative-intend surgery. The cut-off for prognostic stratification was established according to the median overall survival of inoperable, advanced PDAC patients. Small RNA sequencing revealed 44 miRNAs significantly associated with the PDAC patients’ survival after surgery. Out of these, the most promising miRNAs that met the established selection criteria were subjected to further evaluation as prognostic biomarkers in the independent patients’ cohort. Finally, a comparison of miRNA expression level in the validation phase confirmed ability of three miRNAs (miR-99a-5p, miR-200c-3p, and miR-365a-3p) to discriminate PDAC patients with poor outcomes after resection from those with longer survival. Validation confirmed that a high level of miR-99a-5p in pre-operative plasma is associated with better survival of PDAC patients who underwent curative surgery. This finding is indicating its dual role during tumorigenesis and cancer progression. MiR-99a-5p belongs to the twenty most abundant miRNAs in human plasma exosomes, indicating that its appearance in bloodstream is a result of coordinated release from cell in response to different stimuli and could reflect disease status [24]. The role of miR-99a-5p in various cancer types has been described; aberrant expression of this miRNA has been linked with both oncogenic and tumor suppressive function. While results provided by Dhayata et al. imply pro-oncogenic regulatory activity of miR-99a-5p, our observation indicates its protective functioning in the progression of PDAC [25,26]. However, a number of other reports support our observation, indicating miR-99a roles in a suppression of various cancer types. Decreased expression of miR-99a-5p was found to predict worse survival in lung adenocarcinoma [27], cervical cancer [28], and breast cancer [29]. MiR-99a-5p was found to play important tumor-suppressive roles, including inhibition of cell proliferation and tumorigenesis by suppressing mTOR signaling pathway and downregulation of insulin-like growth factor 1 receptor [30], and also inhibition of migration and invasion of cancer cells by decreasing MTMR3 protein (Myotubularin-related protein 3) in oral cancer [31]. 

The second successfully validated miRNA was miR-200c-3p. However, log2(FC) in both discovery and validation cohort was below 1, which could limit its application as a potential biomarker. This miRNA belongs to the miR-200 family and is well described as an epithelial marker in solid tumors, including PDAC. It plays an important role in regulating of epithelial phenotype of tumor cells during both epithelial mesenchymal transition (EMT) and mesenchymal epithelial transition (MET) processes. Results of the metanalysis of Wang et al. demonstrated that low expression of miR-200c in tumor tissue and high expression in serum is correlated with poorer survival in solid tumors [32]. The main targets of or miR-200c are transcriptional repressors, namely E-cadherin and ZEB1 and ZEB2. Insufficient expression of this miRNA led to the loss of epithelial features of cancer cells, thereby, cells acquire the ability to escape from primary localization and further enter to circulation. During reverse process, miR-200c-3p is upregulated, and tumor cells with mesenchymal phenotype acquire epithelial phenotype necessary to final metastatic colonization and formation of macroscopic metastases in the distant organs. The reason for poor prognosis in PDAC patients is rapid disease progression and early dissemination [10]. Therefore, we speculate that higher levels of miR-200c in the blood plasma of PDAC patients with shorter survival results from the presence a non-detectable metastatic disease in the time of tumor resection suggesting diverse roles of miR-200c-3p in different stages of PDAC development.

Finally, we found that a higher level of miR-365a-3p in pre-operative plasma might predict the longer survival of PDAC patients following curative radical resection. To support our observation, reports of Yin et al. note an association between low expression of miR-356a-3p and PDAC progression by in vitro, in vivo, and patient tissue studies [33]. Increased expression of miR-365a-3p inhibits NF-κB activity by downregulating c-Rel and thus reduced the viability of PDAC cells and induced apoptosis. 

A combination of circulating miRNAs may considerably improve cancer diagnosis and prognosis, and some miRNAs panels have already been described as non-invasive biomarkers for PDAC disease. For example, Cao et al. established two plasma miRNAs-based panels with high diagnostic accuracy. The first panel comprising three miRNAs, namely, miR-486-5p, miR-126-3p, and miR-106b-3p, had high accuracy for distinguishing pancreatic cancer (PC) from chronic pancreatitis (CHP) with AUC values of 0.891. Furthermore, panel including 6 microRNAs; miR-486-5p, miR-126-3p, miR-106b-3p, miR-938, miR-26b-3p, miR-1285 accurately discriminate between PC patients and CHP with AUC 0.889. Moreover, both miRNA panels showed higher diagnostic accuracy than CA19-9 (AUC = 0.775) [34]. Most recently, Zou et al. identified a panel of six serum miRNAs (let-7b-5p, miR-192-5p, miR-19a-3p, miR-19b-3p, miR-223-3p, and miR-25-3p) with potential to distinguish PC patients and healthy donors with AUC = 0.910. Besides, the analysis provided in their study revealed that the combination of miRNAs showed higher diagnostic value than the individual miRNA [35]. 

Herein, we showed that three miRNAs-based biomarkers can significantly predict PDAC patients’ survival time after curative surgery. Multiple miRNAs combined are considered as a more superior diagnostic tool than a single miRNA-based test. This thesis can be verified based on the mechanisms of miRNAs in cancer development and progression, whereas a series of miRNAs, rather than a single one, are involved in the pathological process. Moreover, the same miRNAs are deregulated in different types of malignancies suggesting that single miRNAs could not be specific to a cancer type. In this sense, it is reasonable that a combination of miRNAs guarantee that the biomarker is specific to a cancer type. miRNA panels are based on the combination of two miRNAs up to several miRNAs. Nevertheless, an optimal clinical model must have a high sensitivity and specificity and a suitable cost and time-effectiveness.

## 5. Conclusions

In conclusion, we identified miRNAs associated with OS in the pre-operative blood plasma samples of PDAC patients undergoing surgical resection. Following further independent validations, the detection of these miRNA may enable identification of PDAC patients who have no survival benefit from the surgical treatment, which is associated with the high morbidity rates. However, our study suffers from some limitations. As the main limitation, we recognize that the validation part of the study is based on a small prospective sample cohort and further validation with larger sample size is required to validate the efficacy of candidate miRNAs. Although several challenges remain to be addressed, plasma miRNAs can potentially be useful for the prognostic stratification of PDAC patients undergoing curative resection.

## Figures and Tables

**Table 1:** Clinical and histopathological characteristics of pancreatic ductal adenocarcinoma patients.

	Discovery Cohort	Validation Cohort
*n* (Patients)	48	64
Age		
65 years	15	24
>65 years	33	40
Sex		
Male	24	24
Female	24	40
Tumor location		
pancreatic head	38	50
pancreatic body/tail	10	14
pT stage		
T2	0	9
T3	48	55
pN stage		
N0	5	20
N1-2	43	44
pM stage		
M0	48	64
CA19-9		
High	22	30
Low	18	20
NA	8	14
Adjuvant chemotherapy		
Yes	42	44
No	6	20
Poor survival group (median 9, range 4–14 months)	24	14
Good survival group (median 27, range 20–47 months)	24	16

**Table 2:** List of the miRNAs with significantly different levels in blood plasma of patients with good (overall survival (OS) > 20 months) and poor prognosis (OS < 16 months) identified in the discovery phase of the study.

microRNA	BaseMean	log2FC	*p*-Value
**miR-99a-5p**	**200.59**	−**1.19**	**0.001**
**miR−9-5p**	**12.70**	**1.48**	**0.002**
**miR-365a-3p**	**16.54**	−**1.75**	**0.002**
miR-362-5p	3.39	1.07	0.005
miR-627-5p	4.33	−1.26	0.006
**miR-22-3p**	**566.82**	−**0.61**	**0.006**
**miR-885-5p**	**27.65**	−**1.50**	**0.008**
miR-1273h-5p	7.55	0.90	0.009
miR-940	0.89	1.49	0.011
miR-499a-5p	2.38	−1.50	0.011
miR-34c-3p	2.09	2.11	0.012
**miR-200c-3p**	**79.59**	**0.49**	**0.012**
miR-101-5p	1.28	−2.17	0.012
miR-18b-3p	0.66	−2.01	0.014
**let-7e-5p**	**843.66**	**0.54**	**0.014**
**miR-30e-5p**	**3565.64**	−**0.35**	**0.015**
**miR-100-5p**	**99.16**	−**0.97**	**0.015**
**miR-122-5p**	**196,465.98**	−**1.14**	**0.016**
**miR-99b-5p**	**298.30**	**0.45**	**0.019**
let-7b-3p	9.59	−1.02	0.020
let-7f-5p	24,401.75	0.33	0.024
miR-6770-3p	0.64	1.65	0.025
miR-181c-5p	6.71	0.81	0.026
miR-5010-5p	9.20	0.74	0.028
miR-30a-5p	954.09	−0.66	0.030
miR-4676-3p	2.38	1.11	0.030
miR-885-3p	109.35	−1.16	0.030
miR-193b-3p	1.43	−1.69	0.034
miR-12135	3.27	0.95	0.035
miR-1275	5.86	−0.69	0.037
miR-202-3p	2.46	−1.30	0.037
miR-552-5p	2.50	−1.03	0.037
miR-99b-3p	29.24	0.74	0.038
miR-210-3p	14.32	−0.69	0.041
miR-3146	2.11	1.31	0.043
miR-148a-3p	12,136.24	−0.66	0.043
miR-1249-3p	0.83	2.18	0.043
miR-6875-5p	1.63	1.25	0.044
miR-6796-5p	0.68	1.51	0.045
miR-548bc	2.49	1.47	0.046
miR-191-5p	8975.04	0.45	0.047
miR-378a-3p	677.18	−0.63	0.047
miR-224-5p	485.23	−0.66	0.049
miR-96-5p	63.60	−0.78	0.050

Log2FC: Logarithm to the base 2 of fold-change; baseMean: Average of the normalized read numbers; bolded miRNAs were selected for validation in the independent cohort of PDAC patients based on the pre-defined selection criteria. Bold: miRNAs selected for independent validation.

**Table 3:** Results of the validation of miRNA candidates by qPCR in the independent cohort of PDAC patients.

microRNA	NGS Discovery Cohort		qPCR Group Comparison Validation Cohort		qPCR Survival Analysis Validation Cohort
	Log2FC	*p*-Value	Log2FC	*p*-Value	*p*-Value
miR-99a-5p	−1.188	0.001	−1.324	0.03	0.006
miR-365a-3p	−1.752	0.002	−1.39	0.003	0.013
miR-200c-3p	0.493	0.012	0.766	0.04	0.012

Log2FC: Logarithm to the base 2 of fold-change; NGS: Next-generation sequencing; qPCR: Quantitative polymerase chain reaction.

## References

[1] Kleeff J., Korc M., Apte M., La Vecchia C., Johnson C.D., Biankin A.V., Neale R.E., Tempero M., Tuveson D.A., Hruban R.H. (2016). Pancreatic cancer. Nat. Rev. Dis. Primers.

[2] Rawla P., Sunkara T., Gaduputi V. (2019). Epidemiology of Pancreatic Cancer: Global Trends, Etiology and Risk Factors. World J. Oncol..

[3] Poredska K., Kunovsky L., Prochazka V., Dolina J., Chovancova M., Vlazny J., Andrasina T., Eid M., Jabandziev P., Kysela P. (2019). Triple malignancy (NET, GIST and pheochromocytoma) as a first manifestation of neurofibromatosis type-1 in an adult patient. Diag. Pathol..

[4] Neoptolemos J.P., Stocken D.D., Friess H., Bassi C., Dunn J.A., Hickey H., Beger H., Fernandez-Cruz L., Dervenis C., Lacaine F. (2004). A Randomized Trial of Chemoradiotherapy and Chemotherapy after Resection of Pancreatic Cancer. N. Engl. J. Med..

[5] Neoptolemos J.P., Stocken D.D., Smith C.T., Bassi C., Ghaneh P., Owen E., Moore M., Padbury R., Doi R., Smith D. (2009). Adjuvant 5-fluorouracil and folinic acid vs observation for pancreatic cancer: Composite data from the ESPAC-1 and -3(v1) trials. Br. J. Cancer.

[6] Neoptolemos J.P., Palmer D.H., Ghaneh P., E Psarelli E., Valle J.W., Halloran C.M., Faluyi O., A O’Reilly D., Cunningham D., Wadsley J. (2017). Comparison of adjuvant gemcitabine and capecitabine with gemcitabine monotherapy in patients with resected pancreatic cancer (ESPAC-4): A multicentre, open-label, randomised, phase 3 trial. Lancet.

[7] Jang J.-Y., Kang M., Heo J.S., Choi S.H., Choi D.W., Park S.-J., Han S.-S., Yoon D.S., Yu H.C., Kang K.J. (2014). A Prospective Randomized Controlled Study Comparing Outcomes of Standard Resection and Extended Resection, Including Dissection of the Nerve Plexus and Various Lymph Nodes, in Patients with Pancreatic Head Cancer. Ann. Surg..

[8] Farnell M.B., Pearson R.K., Sarr M.G., DiMagno E.P., Burgart L.J., Dahl T.R., Foster N., Sargent D., the Pancreas Cancer Working Group (2005). A prospective randomized trial comparing standard pancreatoduodenectomy with pancreatoduodenectomy with extended lymphadenectomy in resectable pancreatic head adenocarcinoma. Surgery.

[9] Zheng L., Wolfgang C.L. (2015). Which patients with resectable pancreatic cancer truly benefit from oncological resection: Is it destiny or biology?. Cancer Boil..

[10] Rhim A.D., Mirek E.T., Aiello N.M., Maitra A., Bailey J.M., McAllister F., Reichert M., Beatty G.L., Rustgi A.K., Vonderheide R.H. (2012). EMT and Dissemination Precede Pancreatic Tumor Formation. Cell.

[11] Strobel O., Neoptolemos J.P., Jäger D., Büchler M.W. (2018). Optimizing the outcomes of pancreatic cancer surgery. Nat. Rev. Clin. Oncol..

[12] Hong S.B., Lee S.S., Kim J.H., Kim H.J., Byun J.H., Hong S.-M., Song K.-B., Kim S. (2018). Pancreatic Cancer CT: Prediction of Resectability according to NCCN Criteria. Radiology.

[13] Pandiaraja J., Viswanathan S., Antomy T.B., Thirumuruganand S., Kumaresan D.S. (2016). The Role of CA19-9 in Predicting Tumour Resectability in Carcinoma Head of Pancreas. J. Clin. Diagn. Res..

[14] Schanen B.C., Li X. (2011). Transcriptional regulation of mammalian miRNA genes. Genomics.

[15] Jabandziev P., Bohosova J., Pinkasova T., Kunovsky L., Slaby O., Goel A. (2020). The Emerging Role of Noncoding RNAs in Pediatric Inflammatory Bowel Disease. Inflamm. Bowel. Dis..

[16] Galatenko V.V., Galatenko A.V., Samatov T.R., Turchinovich A.A., Shkurnikov M.Y., Makarova J.A., Tonevitsky A.G. (2018). Comprehensive network of miRNA-induced intergenic interactions and a biological role of its core in cancer. Sci. Rep..

[17] Vorvis C., Koutsioumpa M., Iliopoulos D. (2016). Developments in miRNA gene signaling pathways in pancreatic cancer. Futur. Oncol..

[18] Chen X., Ba Y., Ma L., Cai X., Yin Y., Wang K., Guo J., Zhang Y., Chen J., Guo X. (2008). Characterization of microRNAs in serum: A novel class of biomarkers for diagnosis of cancer and other diseases. Cell Res..

[19] Mitchell P.S., Parkin R.K., Kroh E.M., Fritz B.R., Wyman S.K., Pogosova-Agadjanyan E.L., Peterson A., Noteboom J., O’Briant K.C., Allen A. (2008). Circulating microRNAs as stable blood-based markers for cancer detection. Proc. Natl. Acad. Sci. USA.

[20] Weber J.A., Baxter D.H., Zhang S., Huang D.Y., Huang K.H., Lee M.-J., Galas D.J., Wang K. (2010). The MicroRNA Spectrum in 12 Body Fluids. Clin. Chem..

[21] McAlexander M.A., Phillips M.J., Witwer K.W. (2013). Comparison of Methods for miRNA Extraction from Plasma and Quantitative Recovery of RNA from Cerebrospinal Fluid. Front. Genet..

[22] Marabita F., De Candia P., Torri A., Tegnér J., Abrignani S., Rossi R.L. (2015). Normalization of circulating microRNA expression data obtained by quantitative real-time RT-PCR. Brief. Bioinform..

[23] Karasek P., Gablo N., Hlavsa J., Kiss I., Vychytilova-Faltejskova P., Hermanová M., Kala Z., Slaby O., Prochazka V. (2018). Pre-operative Plasma miR-21-5p Is a Sensitive Biomarker and Independent Prognostic Factor in Patients with Pancreatic Ductal Adenocarcinoma Undergoing Surgical Resection. Cancer Genom. Proteom..

[24] Huang X., Yuan T., Tschannen M., Sun Z., Jacob H.J., Du M., Liang M., Dittmar R.L., Liu Y., Liang M. (2013). Characterization of human plasma-derived exosomal RNAs by deep sequencing. BMC Genom..

[25] Stroese A.J., Ullerich H., Koehler G., Raetzel V., Senninger N., Dhayat S.A. (2018). Circulating microRNA-99 family as liquid biopsy marker in pancreatic adenocarcinoma. J. Cancer Res. Clin. Oncol..

[26] Dhayat S.A., Mardin W.A., Seggewiß J., Ströse A.J., Matuszcak C., Hummel R., Senninger N., Mees S.T., Haier J. (2015). MicroRNA Profiling Implies New Markers of Gemcitabine Chemoresistance in Mutant p53 Pancreatic Ductal Adenocarcinoma. PLoS ONE.

[27] Song Y., Dou H., Wang P., Zhao S., Wang T., Gong W., Zhao J., Li E., Tan R., Hou Y. (2014). A novel small-molecule compound diaporine A inhibits non-small cell lung cancer growth by regulating miR-99a/mTOR signaling. Cancer Biol. Ther..

[28] Wang L., Chang L., Li Z., Gao Q., Cai D., Tian Y., Zeng L., Li M. (2014). MiR-99a and -99b inhibit cervical cancer cell proliferation and invasion by targeting mTOR signaling pathway. Med. Oncol..

[29] Hu Y., Zhu Q., Tang L. (2014). MiR-99a Antitumor Activity in Human Breast Cancer Cells through Targeting of mTOR Expression. PLoS ONE.

[30] Cheng H., Xue J., Yang S., Chen Y., Wang Y., Zhu Y., Wang X., Kuang D., Ruan Q., Duan Y. (2017). Co-targeting of IGF1R/mTOR pathway by miR-497 and miR-99a impairs hepatocellular carcinoma development. Oncotarget.

[31] Kuo Y.-Z., Tai Y.-H., Lo H.-I., Chen Y.-L., Cheng H.-C., Fang W.-Y., Lin S.-H., Yang C.-L., Tsai S.-T., Wu L.-W. (2013). MiR-99a exerts anti-metastasis through inhibiting myotubularin-related protein 3 expression in oral cancer. Oral Dis..

[32] Wang H., Shen J., Jiang C.-P., Liu B. (2014). How to Explain the Contradiction of microRNA 200c Expression and Survival in Solid Tumors?: A Meta-analysis. Asian Pac. J. Cancer Prev..

[33] Yin L., Xiao X., Georgikou C., Yin Y., Liu L., Karakhanova S., Luo Y., Gladkich J., Fellenberg J., Sticht C. (2019). MicroRNA-365a-3p inhibits c-Rel-mediated NF-kappaB signaling and the progression of pancreatic cancer. Cancer Lett..

[34] Cao Z., Liu C., Xu J., You L., Wang C., Lou W., Sun B., Miao Y., Liu X., Wang X. (2016). Plasma microRNA panels to diagnose pancreatic cancer: Results from a multicenter study. Oncotarget.

[35] Zou X., Wei J., Huang Z., Zhou X., Lu Z., Zhu W., Miao Y. (2019). Identification of a six-miRNA panel in serum benefiting pancreatic cancer diagnosis. Cancer Med..

